# Patterns of Exposure and Infection with Microparasites in Iberian Wild Carnivores: A Review and Meta-Analysis

**DOI:** 10.3390/ani11092708

**Published:** 2021-09-16

**Authors:** Javier Millán, Daniel J. Becker

**Affiliations:** 1 Instituto Agroalimentario de Aragón-IA2, Universidad de Zaragoza-CITA, 50013 Zaragoza, Spain; 2 Fundación ARAID, Avda. Ranillas 1, 50018 Zaragoza, Spain; 3 Facultad de Ciencias de la Vida, Universidad Andres Bello, Santiago 8320000, Chile; 4 Department of Biology, University of Oklahoma, Norman, OK 73019, USA; danbeck@ou.edu

**Keywords:** Carnivora, conservation, Europe, Mediterranean, One Health

## Abstract

**Simple Summary:**

Carnivores are a relevant taxon in the field of wildlife diseases due to their ecological and behavioral traits, and they are key hosts in the epidemiology of infectious diseases in the fields of public, pet, and livestock health. Conversely, their conservation is also directly threatened by disease outbreaks. The Iberian Peninsula, located in the southwest of the Eurasian continent, hosts a diverse assemblage of carnivores, including 18 species belonging to seven different families. In this article, we review the state of the art in the epidemiology of infectious diseases in wild carnivores in Spain and Portugal and use meta-analytic and comparative methods to derive insights into how sampling effort, pathogen richness, infection prevalence, and prevalence of antibodies vary across carnivore taxa and Iberian geography. We also identify important pitfalls and future perspectives for research. Our understanding of infectious diseases in Iberian wild carnivores has significantly advanced in the last twenty years, but there is a lack of longitudinal studies of infectious disease in Iberian carnivores.

**Abstract:**

We use a suite of meta-analytic and comparative methods to derive fundamental insights into how sampling effort, pathogen richness, infection prevalence, and seroprevalence vary across Carnivora taxa and Iberian geography. The red fox was the most studied species, the wolf and Iberian lynx were disproportionally studied, and the Arctoidea were understudied. Sampling effort was higher in Mediterranean areas, but central Spain showed the higher pathogen richness. Excluding studies analyzing fecal samples, 53 different pathogens have been detected in Iberian carnivores, including 16 viruses, 27 bacteria, and 10 protozoa but no fungi. Sampling effort and pathogen diversity were generally more similar among closely related carnivore species. Seropositivity to viruses was lower and higher in the Mustelinae and the Canidae, respectively, and seropositivity to protozoa was higher in both taxa. Canine distemper virus exposure was greatest in canids and mustelids. Carnivore protoparvovirus-1 exposure was greatest in the Atlantic regions, and the Felidae and the Musteloidea had lower infection prevalence. A subclade of the Mustelidae had a greater prevalence of *Leishmania* infection. We observed no relationships between host phylogenetic distance and pathogen sharing among species. Lastly, we identify important research pitfalls and future directions to improve the study of infectious disease in Iberian wild carnivore communities.

## 1. Introduction

Carnivores are a relevant taxon in the field of wildlife disease due to their ecological and behavioral traits. Carnivores are closely related to the two most widespread domestic pets, the dog and cat, and are susceptible to almost all of their pathogens [1]. Carnivores occupy high trophic levels of food webs, which constantly exposes them to a vast number of pathogens from their prey [2]. Some carnivores are also social species, which favors the transmission of directly transmitted pathogens [3]. Carnivores also display intrinsic aggressive behavior, both intra and interspecific, which is used by many pathogens as a transmission route [4]. Many carnivores are also dietary generalists and can thrive in human-dominated landscapes, where they may eat garbage, leftovers, livestock and fowl, aborted animal fetuses due to parasitic or infectious diseases, etc. [5], putting them into contact with several pathogens. Their approach to human dwellings also increases contact with domestic dogs and cats and opportunities for spillover [6]. In the current scenario of global change and increased human encroachment into natural habitats, these opportunities are probably higher than ever before and will continue to increase.

Carnivores are key hosts in the epidemiology of infectious diseases in the fields of public, pet, and livestock health, and, conversely, their conservation is directly threatened by disease outbreaks [7]. Moreover, and from the One Health perspective, many of these pathogens are relevant for the health of more than one of these taxonomic groups. For example, the red fox (*Vulpes vulpes*) acts as a natural reservoir for rabies virus in some European regions, which poses a risk for the health of humans, livestock, pets, and threatened wildlife [8]. Other relevant pathogens that include carnivores among their hosts (either as reservoirs or recipient hosts) in Europe include distemper viruses [9], protoparvoviruses [10], feline retroviruses [11], and *Leishmania infantum* [12].

The Iberian Peninsula, located in the southwest of the Eurasian continent, hosts a diverse assemblage of carnivores including 18 species belonging to seven different families [13,14]. Iberia holds the largest wolf (*Canis lupus*) population of western Europe, the last Iberian lynx (*Lynx pardinus*) populations, two isolated brown bear (*Ursus arctos*) populations, up to nine mustelid species, and some introduced viverrid, herpestid, and procyonid species, either historically by Romans and Arabs, or recently (Table 1). Whereas almost no research was conducted in the field of wildlife diseases in the 20th century (with the exception of macroparasites, such as ectoparasites and helminths), carnivores started to attract the interest of the Iberian researchers around the turn of the century. In consequence, during the last twenty years, information about the prevalence of certain pathogens has increased substantially.

However, this information has been generated by diverse research groups and remains fragmented in the literature. This is why we considered it timely to review the state of the art in the epidemiology of infectious diseases (specifically, microparasites including viruses, bacteria, fungus, and protozoa) in free-living carnivores in the Iberian Peninsula and the Balearic Islands. Here we use a suite of meta-analytic and comparative methods to derive fundamental insights into how sampling effort, pathogen richness, infection prevalence (i.e., the prevalence of active infections), and seroprevalence (i.e., the prevalence of past infections) varied across carnivore taxa and Iberian geography. More specifically, we aimed to identify the most frequently studied hosts and pathogens, differences in pathogen exposure or infection depending on the host taxon, trends in pathogen sharing between taxa, geographical variation in prevalence and seroprevalence and, most importantly, research gaps. Lastly, we aimed to provide researchers with future directions to improve the knowledge on infectious disease in Iberian wild carnivore communities. 

## 2. Materials and Methods

### 2.1. Search Strategy and Study Selection

We performed a systematic search following the PRISMA (Preferred Reporting Items for Systematic Reviews and Meta-Analyses) guideline for systematic reviews [15]. Our search included the databases Google Scholar, PubMed, Web of Science Core Collection, and Scopus, using the following query: ((“wild carnivore” OR all the scientific and English common names of all the carnivore species) AND (Iberian Peninsula OR Portugal OR Spain OR Andorra) AND (disease OR pathogen OR parasite OR virus OR bacteria OR fungus OR protozoa)). References identified by the search were screened for inclusion criteria and relevance to the review question by two of the co-authors (JM and DJB). Discrepancies were resolved by consensus. Studies were selected using the following inclusion criteria: studies published from inception to 31 January 2021, and that investigated microparasites (i.e., virus, bacteria, fungus, and protozoa) in free-living carnivores. Studies based solely on fecal samples, studies of clinical cases in a single individual, grey literature, and conference abstracts were excluded from our quantitative analyses. Some studies not included in the quantitative analysis (i.e., studies on fecal samples, case reports, Ph.D. theses) are however later discussed in the manuscript. Studies in the Balearic Islands were included because of the similar ecological characteristics of these islands with the Iberian Peninsula and because all the carnivore species present there were historically introduced first from Iberia.

### 2.2. Quantitative Data Analysis

**Figure 1:**
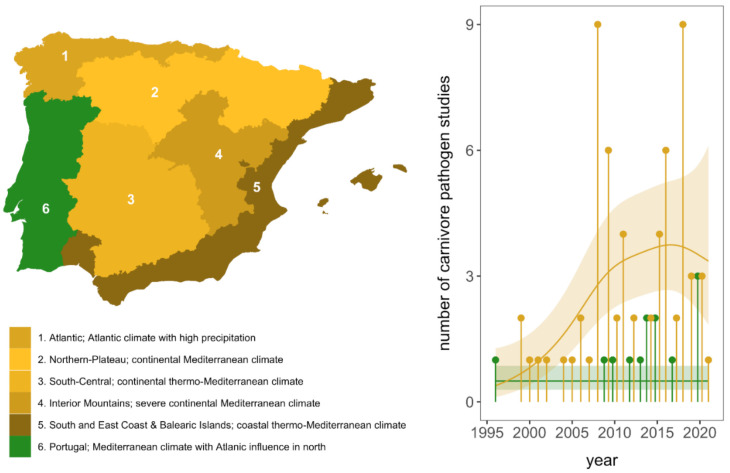
(**Left**): Bioregions of Iberia and their associated climatic conditions. (**Right**): Number of studies of microparasites of carnivores in the Iberian Peninsula per year and country (yellow = Spain, green = Portugal). Points are jittered to reduce overlap and are overlaid with the fitted mean and 95% confidence interval band from our GAM of study effort over time. See Table 1 for details about carnivore species presence on the different bioregions.

For our analyses, seventy-six articles were finally selected. From all studies, we extracted the number of sampled and positive animals per carnivore species, publication year, sampled region and bioregion, pathogen, and detection method. To assess temporal trends in research effort, we fit a generalized additive model (GAM) with the number of studies per year as a Poisson-distributed response and used country, a smoothed term for year, and their interaction as predictors using the *mgcv* package in R [16]. For regional analyses, the Spanish Wildlife Disease Surveillance Scheme defines five bioregions across Spain with distinct habitat and climatic conditions [17], to which we included an additional region to encompass Portugal (Figure 1). We distinguished between direct (e.g., PCR, culture, microscopy; infection prevalence) and indirect assays (e.g., enzyme-linked immunosorbent assays, immunofluorescence assays; seroprevalence) and included taxonomic information for hosts and pathogens. This resulted in 711 host-pathogen-region-method associations. Most studies (78%) contributed multiple records. We matched our 18 carnivore species against a mammal phylogeny [18] using the *ape* package [19].

We used these data to assess taxonomic and geographic patterns in our four response variables (i.e., sampling effort, pathogen richness, infection prevalence, and seroprevalence). For sampling effort, we used a generalized linear model (GLM) with the number of studies per unique combination of carnivore species and region as a Poisson-distributed response variable (*n* = 135) and host family and bioregion as predictors. For pathogen richness, we used another GLM with the number of pathogens modeled using a negative binomial distribution with the *MASS* package [20]. For our GLM of pathogen richness, we included the above number of studies to control for sampling effort. For infection prevalence (*n* = 421) and seroprevalence (*n* = 251), we included host family, bioregion, and their interaction in phylogenetic meta-analysis models. We used the *metafor* package to calculate Freeman–Tukey double arcsine transformed proportions of positive carnivores and sampling variances [21]. Given moderate phylogenetic relationships between carnivores (mean *r*, excluding the diagonal = 0.32; Figure S1), we included random effects for species and phylogeny as well as study- and observation-level random effects [22]. For each model, we assessed fit using Nagelkerke’s *R*^2^ [23] for GLMs or, for meta-analysis models, as the proportional reduction in the summed variance components compared against those from an intercept-only model [24]. We adjusted for the inflated false-discovery rate in post-hoc comparisons using the *emmeans* and *multcomp* packages [25].

To assess phylogenetic patterns in our response variables we used the *caper* package to estimate phylogenetic signal to data aggregated per carnivore species (Pagel’s λ) [26,27]. Next, we applied a graph partitioning algorithm, phylogenetic factorization, to more flexibly identify any carnivore clades across taxonomic levels that differ in each response variable. With a standardized taxonomy [18], we used the *phylofactor* package to partition studies, pathogen richness, infection prevalence, and seroprevalence per species in a series of GLMs [28]. We modeled these variables with Poisson, negative binomial, and binomial distributions; for pathogen richness, we included the number of studies as a covariate. We determined the number of significant clades using Holm’s sequentially rejective test with a 5% family-wise error rate.

Because data spanned multiple pathogens from different taxonomic groups, we repeated these analyses (where possible, given the sample size) for viruses, bacteria, and protozoa. We also separately analyzed data for the three most frequently studied pathogens: canine distemper virus (CDV, *n* = 97), carnivore protoparvovirus-1 (CPV-1, *n* = 97), and *L. infantum* (*n* = 55). For taxonomic and bioregional analyses of positivity for these three pathogens, we pooled data from direct and indirect detection methods as a proxy for exposure.

Lastly, we assessed phylogenetic and regional patterns in pathogen sharing. Similar to prior analyses [29], we considered the presence of the same pathogen in two species to indicate sharing, while acknowledging that cross-species transmission is better captured by finer-scale genotype variation within pathogen species [30]. For our data on pathogen detections (*n* = 332), we used the *igraph* package to build adjacency networks for each bioregion, where nodes represent species and edges represent shared pathogens [31]. We used the ape package to derive phylogenetic distance among carnivore species. We then fit a GAM with *mgcv* to test how pathogen sharing varied across bioregions and with phylogenetic relatedness. We modeled pathogen sharing as a Poisson response, given common pathogen sharing after excluding identical species pairs (i.e., we observed pathogen sharing in 17–100% of species pairs per bioregion, *x* = 0.74). Our GAM included bioregion, a smoothed effect of phylogenetic distance, and their interaction; we also included a smooth term for the total sample size for each species pair to account for sampling effort.

## 3. Results

### 3.1. Literature Patterns

Seventy-six articles fulfilled our selection criteria, of which 13 were performed in Portugal and all the remainder, in Spain. No transboundary article was published, and no article was retrieved from Andorra. Except for one article published in Portugal in 1996, publications began in 1999 in Spain at a rate of one/two articles per year and remained constant until 2008–2009, when the number of studies increased markedly in Spain and started in Portugal (Figure 1). This increase in Spain was distinct from temporal publication trends for Portugal, which showed no increase (GAM: *χ*^2^ = 15.22, *p* < 0.001).

**Figure 2:**
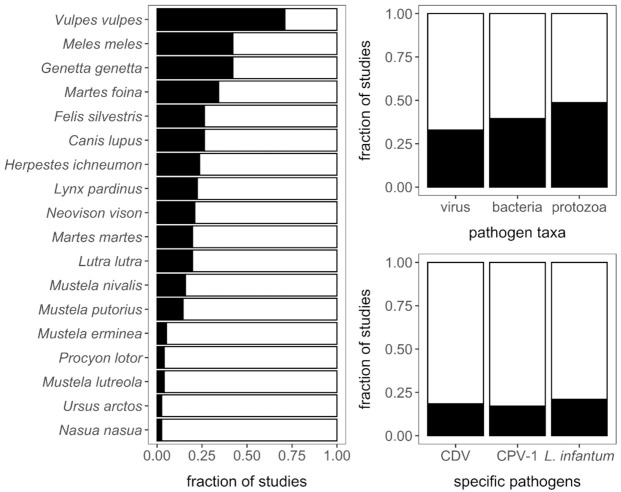
Composition of the 76 included Iberian carnivore microparasite studies according to host species, pathogen taxa, and the three most common pathogens.

All 18 carnivore species in Iberia were studied at least once for microparasites (Figure 2). The red fox was included in 71% of studies, followed by the Eurasian badger (*Meles meles*; 42%) and the common genet (*Genetta genetta*; 42%). The wolf and the Iberian lynx were disproportionally studied (26% and 22%) when considering their small population sizes. Small mustelids and introduced procyonids were infrequently analyzed. The brown bear and the South American coati (*Nasua nasua*) were only included in one study each.

Our review included studies of 53 different pathogens, including 16 viruses, 27 bacteria, and 10 protozoa; no fungal pathogens were identified. Galván-Díaz et al. [32] detected the fungal agent *Enterocytozoon bieneusi* in fox fecal samples; given our inclusion criteria, this study was not included in our analyses. Most articles (*n* = 65) dealt with only a single pathogen taxon. Thirty-three percent of articles included viruses, 39% included bacteria, and 49% included protozoa (Figure 2). The most studied pathogens were CDV and CPV-1 among the viruses, *Mycobacterium bovis* and pathogenic *Leptospira* among the bacteria, and *Leishmania infantum* among the protozoa (Figure 2).

### 3.2. Sampling Effort and Pathogen Richness

**Figure 3:**
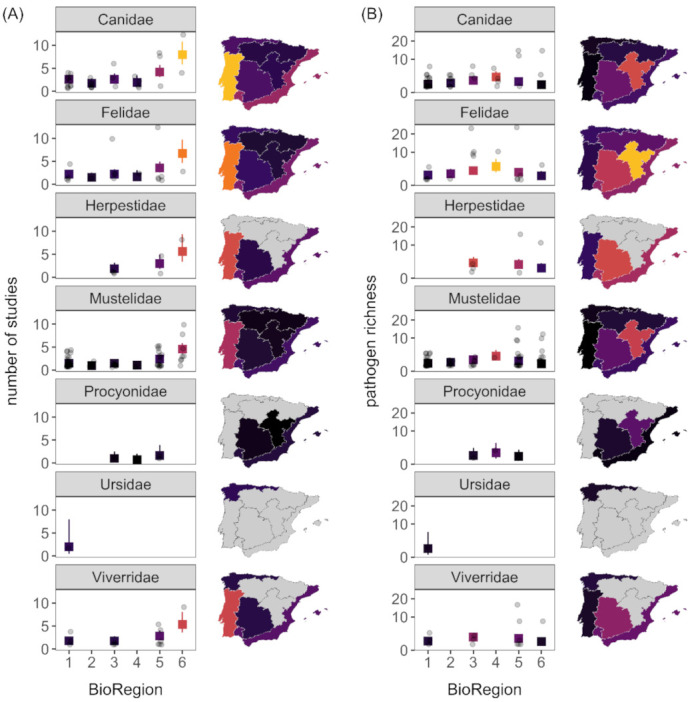
Sampling effort (i.e., number of studies; **A**) and pathogen richness (**B**) as a function of carnivore family and bioregion. Predicted means and 95% confidence intervals from GLMs are shown with raw data. Choropleth maps display the predicted mean responses for bioregion averaged across carnivore family. Modeled results for pathogen richness display predictions after adjusting for mean sampling effort, and the vertical axis uses a modulus transformation to accommodate the skewed distribution.

The number of studies per carnivore species and region varied by host family (*χ*^2^ = 18.07, *p* < 0.01) but more so by bioregion (*χ*^2^ = 63.16, *p* < 0.001). These predictors explained 53% of the variation in sampling effort. Across carnivore families, sampling effort was only greater in the Canidae compared to the Mustelidae (Table S1). Bioregion 5 and 6 had greater sampling effort than other bioregions (Figure 3A, Table S2). Pathogen richness was instead driven by bioregion (*χ*^2^ = 17.56, *p* < 0.01), after adjusting for sampling effort (*β* = 0.26, *z* = 12.10, *p* < 0.001). Bioregion and sampling effort explained 93% of the variation in pathogen diversity, and host families did not differ in pathogen richness (*χ*^2^ = 6.69, *p* = 0.35; Figure 3B, Table S3). After accounting for sampling effort, bioregion 4 had more pathogens than bioregions 1 and 6, whereas bioregions 2, 3, and 5 had intermediate pathogens (Figure 3B, Table S4).

### 3.3. Infection Prevalence and Seroprevalence

**Figure 4:**
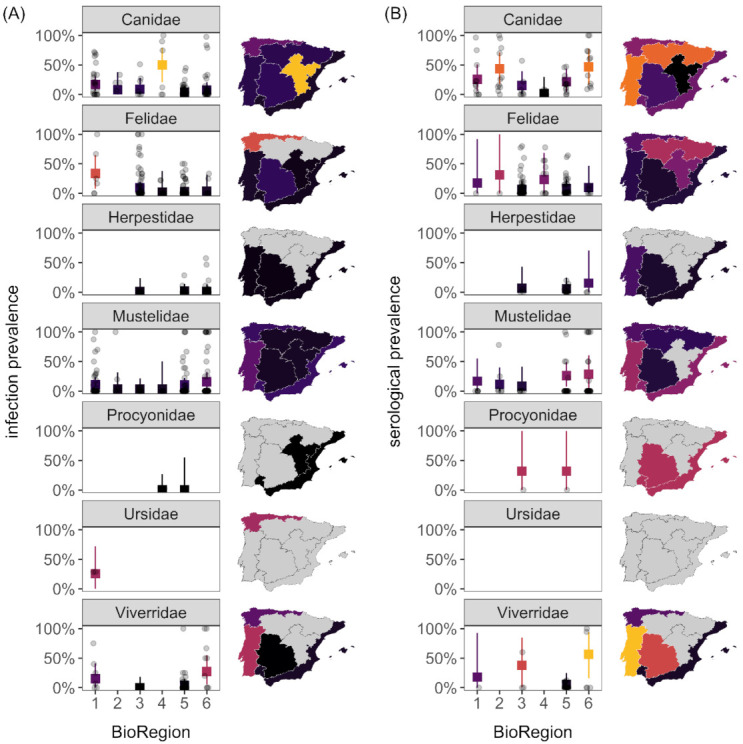
Infection prevalence (**A**) and seroprevalence (**B**) according to carnivore family and bioregion. Predicted means and 95% confidence intervals from meta-analysis models are shown with raw data. Choropleth maps display the predicted mean responses for bioregion averaged across carnivore family.

Across our 44 pathogens assessed by direct detection, the interaction between carnivore family and bioregion was weak (*Q*_15_ = 20.45, *p* = 0.16), and the meta-analysis model explained 5.5% of variation in infection prevalence. Infection prevalence was predicted to be significantly greatest (e.g., ≥20%, with 95% confidence intervals above 0%) for canids sampled in bioregion 4, felids sampled in bioregion 1, and viverrids sampled in bioregion 6 (Figure 4A, Table S5). For the 30 pathogens assessed by indirect detections, the interaction between carnivore family and bioregion was not supported (*Q*_15_ = 14.52, *p* = 0.49), and the meta-analysis model explained 14.4% of the variation in seroprevalence. Seroprevalence did not vary by family (*Q*_5_ = 1.70, *p* = 0.89) but differed weakly by bioregion (*Q*_5_ = 9.14, *p* = 0.10). Seroprevalence was predicted to be significantly greatest in bioregions 1 and 2 for canids; bioregion 3 for viverrids; bioregion 5 for canids or mustelids; and bioregion 6 for canids, viverrids, or mustelids (Figure 4B, Table S6).

### 3.4. Phylogenetic Patterns

**Figure 5:**
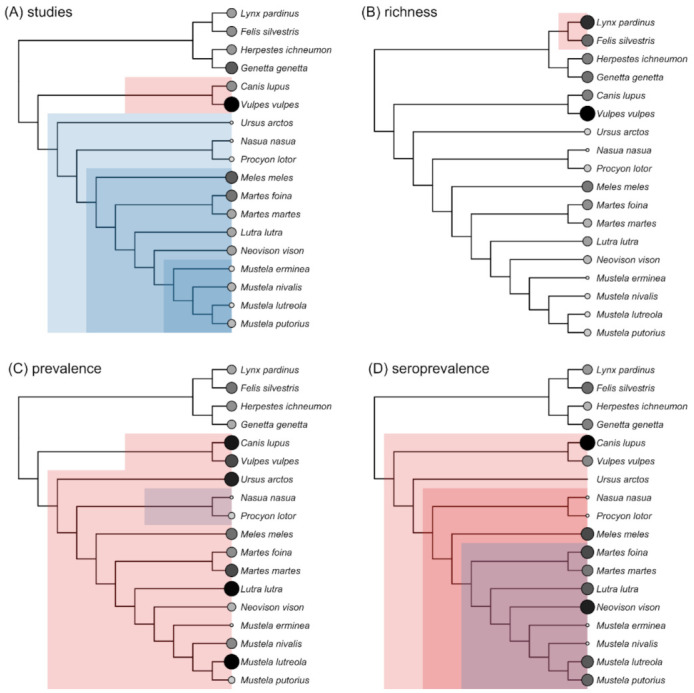
Taxonomic patterns in sampling effort (**A**); pathogen richness, after adjusting for sampling effort (**B**); total infection prevalence (**C**); and total seroprevalence (**D**) across the Iberian carnivore phylogeny. Points are scaled and colored by each response variable. Shading denotes carnivore clades with greater (red) or lesser (blue) of each response variable compared to the paraphyletic remainder as identified by phylogenetic factorization.

When aggregating pathogen data to carnivore species, we observed a strong phylogenetic signal in sampling effort (λ = 0.86) and pathogen richness (λ = 1) but not infection prevalence or seroprevalence (λ = 0). This indicates sampling effort and pathogen diversity were more similar among closely related carnivores. However, phylogenetic factorization identified taxonomic patterns in all response variables that did not strictly correspond to order (Table S7). We identified four clades with distinct numbers of pathogen studies in Iberia: the infraorder Arctoidea (*x* = 11.75), the family Mustelidae (*x* = 14.89), and the genus Mustela (*x* = 7.5) all had lower sampling effort, whereas the Canidae (*x* = 36) had greater sampling effort (Figure 5A). For pathogen richness, and after accounting for sampling effort, the Felidae had more pathogens (*x* = 19) than remaining carnivores (*x* = 7.43; Figure 5B). The Canidae (*x* = 0.26) and a subclade of the Caniformes (*x* = 0.17) had greater infection prevalence, whereas the Procyonidae had lower infection prevalence (*x* = 0.01; Figure 5C). The Mustelidae (*x* = 0.21) and Canidae (*x* = 0.30) had greater seroprevalence, whereas the subfamily Mustelinae had lower seroprevalence (*x* = 0.17; Figure 5D).

### 3.5. Pathogen-Specific Analyses

A number of studies displayed a strong phylogenetic signal for viruses (λ = 0.90) and protozoa (λ = 0.63) but had a weaker phylogenetic signal for bacteria (λ = 0.51). For our three most common pathogens, the phylogenetic signal was stronger for CDV (λ = 0.82) and CPV-1 (λ = 0.90) than *L. infantum* (λ = 0.45). Phylogenetic factorization showed that the number of studies remained consistently lower in the infraorder Arctoidea for bacteria (*x* = 4), viruses (*x* = 3.42), and protozoa (*x* = 4.75) compared to other carnivores. We found similar patterns for CDV and CPV-1; both had fewer studies in the Arctoidea (*x* = 1.83 and 2.58, respectively). For *L. infantum*, however, the Canidae had a greater sampling effort (*x* = 7).

For viruses, bacteria, and protozoa, infection prevalence and seroprevalence varied across carnivore families and bioregions to different degrees (Table S8). For infection prevalence, virus and protozoan positivity varied by bioregion, whereas bacteria positivity did not, and none of the three pathogen groups varied by host family. Virus prevalence was greatest in bioregion 1, whereas protozoan prevalence was greatest in bioregions 1, 3, and 4 (Figure S2, Table S9). Seroprevalence did not vary by host family or bioregion for the three pathogen taxa (Figure S3, Table S10). When considering exposure (i.e., presence of either antigen or antibodies) for our specific pathogens, positivity varied weakly by host family but not bioregion for CDV and weakly for bioregion but not host family for CPV-1; *Leishmania* infantum exposure did not vary by host family nor bioregion (Table S11). CDV exposure was greatest in canids and mustelids, whereas CPV-1 exposure was greatest in bioregions 1 and 2 (driven by canids; Figure S4).

When aggregating infection prevalence and seroprevalence data to carnivore species, we observed no phylogenetic signal for bacteria, viruses, or protozoa (all λ = 0). However, for our most common pathogens, we observed a strong phylogenetic signal for CPV-1 prevalence (λ = 1) and seroprevalence (λ = 0.59) as well as CDV seroprevalence (λ = 0.87). Other pathogen–detection method combinations had either no phylogenetic dependence (i.e., λ = 0) or insufficient sample sizes.

**Figure 6:**
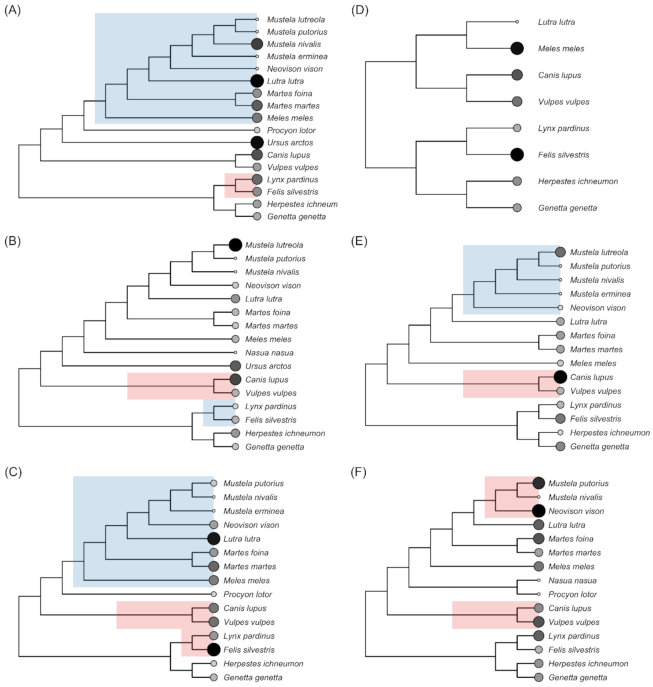
Taxonomic patterns in infection prevalence (**A**–**C**) and seroprevalence (**D**–**F**) for bacteria (top), viruses (middle), and protozoa (bottom) across the Iberian carnivore phylogeny. Points are scaled and colored by the total proportion of positive hosts. Shading denotes clades with greater (red) or lesser (blue) of each response compared to the paraphyletic remainder as identified by phylogenetic factorization.

Phylogenetic factorization found taxonomic patterns in infection prevalence and seroprevalence for most pathogen taxa (Figure 6 and Table S12) and our specific pathogens (Figure S5 and Table S13). For bacteria, prevalence was greatest in the Felidae and lower in the Mustelidae. For viruses, prevalence was lower in the Felidae and greater in the Canidae. Protozoa had greater prevalence in the Canidae and Felidae and were less common in the Mustelidae. For seroprevalence, we identified no taxonomic patterns for bacteria. Viruses had lower seropositivity in the Mustelinae and higher seropositivity in the Canidae. For protozoa, seropositivity was greater in both the Mustelinae and the Canidae. For CDV, we identified no taxonomic patterns in prevalence. Phylogenetic factorization independently confirmed that the Canidae had greater seroprevalence than any other clade. For CPV-1, the Felidae and Musteloidea had lower infection prevalence, but we did not find taxonomic patterns in seroprevalence. Lastly, for *L. infantum*, a subclade of the Mustelidae (genera *Martes*, *Mustela*, and *Neovison*) had greater prevalence. Insufficient species-level data prevented identifying taxonomic patterns in *L. infantum* seroprevalence.

### 3.6. Pathogen Sharing

**Figure 7:**
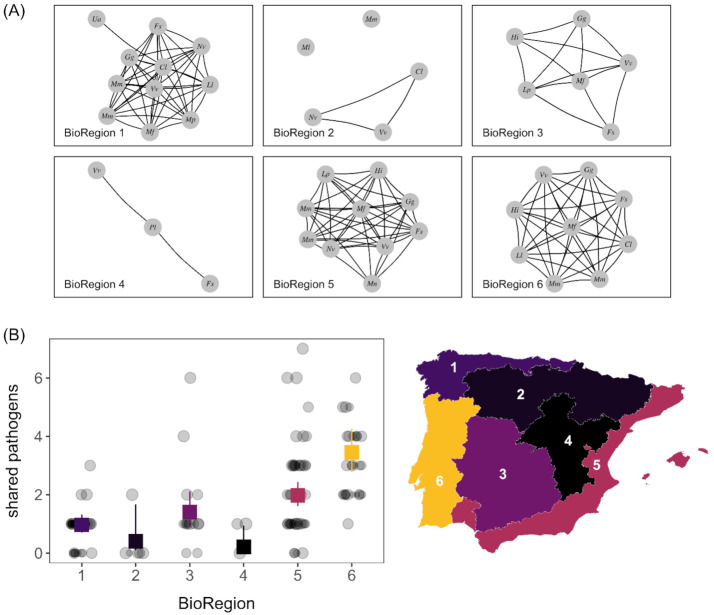
Bioregional patterns of pathogen sharing among Iberian carnivores. (**A**) Nodes in pathogen sharing networks denote host species (abbreviated by their Latin binomial) whereas edges represent two nodes infected by the same pathogen. Networks are stratified by bioregion. (**B**) Modeled relationship (fitted values and 95% confidence intervals from the GAM) between bioregion and pathogen sharing frequency, with raw data overlaid. Maps display GAM-predicted fitted means.

Pathogen sharing was heterogeneous across Iberia (Figure 7A). Our GAM explained 50.8% of the deviance in pathogen sharing frequency (Table S14). We observed no overall or regional relationships between host phylogenetic distance and pathogen sharing, indicating generally common pathogen exchange across carnivores (Figure S6). After accounting for sampling effort, bioregion better predicted pathogen sharing, which was more common in bioregions 3, 5, and 6 (Figure 7B and Table S15).

## 4. Discussion

### 4.1. Publication Trends

The number of scientific publications focused on microparasites in Iberian carnivores increased at the end of the 20th century. This trend has been observed in other European countries [33]. The first article on this topic was published in Portugal in 1996, in which exposure to *Leishmania infantum* was investigated in five red foxes [34]. Studies steadily started to be published in Spain from 2000 onwards, with a sharp and significant increase starting around 2008, when researchers in Portugal started to publish their contributions. Regrettably, no transboundary study has yet been done.

### 4.2. Quantitative Analyses

Sampling effort for microparasites in carnivores was highest in bioregions 5 and 6. Unfortunately, some studies in Spain did not provide the exact origin of their samples, which excluded such research from this analysis and may have affected our results. Most studies conducted in bioregion 5 were performed in Doñana, a protected area in southern Spain, which is home to a metapopulation of the Iberian lynx. On the other hand, bioregion 6 corresponds with all of Portugal, and including a whole country as a unit of analyses may have biased sampling effort results.

Among carnivores, the canids received the most research effort, likely because the red fox is the most abundant species in Iberia, is often considered a reservoir for many pathogens, and can be legally hunted in both countries (and thus access to samples is easier). The wolf is also a species of particular interest for conservation biologists, and their close phylogenetic relationship with domestic dogs facilitates pathogen sharing. In contrast, the Arctoidea, except for the abundant stone marten, had a lower research effort, which is concerning because some of these species are endangered (e.g., the brown bear and European mink) or are experiencing population declines (e.g., the Western polecat) [35]. Lower sampling effort is particularly evident for the smallest representatives of the family (genus *Mustela*). Presumably, their small size, difficulty to detect, identify, or capture; or a possible lack of interest from researchers may have caused this sampling bias. The brown bear has also been understudied and was only sampled in bioregion 1; the reduced population size of this species and its protected status is the most likely cause of this. Remarkably, no data are available for bears from the small, isolated Pyrenean population (bioregion 2).

After accounting for sampling effort, pathogen richness was greatest in bioregion 4 and in felids. Studies in bioregion 4 included many pathogens that were only investigated in this part of Iberia, such as hantavirus, lymphocytic choriomeningitis virus, *Borrelia burdogferi*, *Rickettsia slovaca*, and *Rickettsia typhi*. Higher pathogen richness in felids may have been influenced by the fact that the most endangered felid in the world, the Iberian lynx, has been heavily studied for pathogens during the last twenty years.

When considering all pathogens, infection prevalence and seroprevalence showed relatively weak variation across carnivore families and bioregions. Using a more flexible phylogenetic analysis, canids and procyionids generally showed greater and lower infection prevalence, respectively, than other carnivores. Procyonid populations in Spain originated from escapes or releases from individuals kept as pets [36]. We expect these animals had reduced contact with pathogens compared to truly free-living animals. Nevertheless, once these populations are well established in Spain, they can constitute an important reservoir for pathogens, as observed elsewhere in Europe [37]. A similar scenario can be expected for the coati, which was recently introduced in Mallorca Island. Canids (and mustelids) also had greater seroprevalence. Being close relatives with the domestic dog puts wild canids at a higher risk of pathogen transmission. Both the fox and wolf have been found to be exposed to the main dog pathogens. The red fox is also the most abundant and widely distributed species and should have higher rates of intra and inter-specific contacts. Lastly, canids are more prone to scavenging behavior than other carnivores, which may further contribute to bioaccumulation of pathogens during feeding. For broad pathogen taxa, felids had greater bacterial and protozoan prevalence but lower viral prevalence, whereas canids had greater viral and protozoan prevalence. One explanation could stem from the high pathogenicity of many viruses detected in felids such as feline leukemia virus (FeLV) and feline immunodeficiency virus, which thus present with low prevalences. Other explanations could include different life histories between felines and canines, or even the preferences of researchers resulting in some degree of bias.

### 4.3. Highlights of Wild Carnivore Diseases in Iberia

Perhaps the most paradigmatic group of studies have orbited around the conservation of the Iberian lynx. At the beginning of the century, this species was at the edge of extinction [38]. With this context and the extinction of the black-footed ferret (*Mustela nigripes*) from the wild in mind [39], possible disease outbreaks in the remnant populations were considered direct threats for lynx survival. A large-scale study was performed to determine the prevalence of infectious and parasitic agents in Iberian lynx and to identify potential reservoir hosts [40]. This report and subsequent publications showed that the lynx had little acquired immunity against most of the studied pathogens. Further, FeLV, protoparvovirus, and CDV, harbored by sympatric carnivores, as well as *Mycobacterium bovis* and Aujeszky disease virus (ADV, aka SuHV-1) hosted by ungulates, were identified as the most imminent threats for the Iberian lynx [40,41,42,43,44]. Indeed, in late 2006–early 2007, an outbreak of FeLV stemming from domestic cats killed about two thirds of the lynx in one of the core Iberian metapopulations [45]. After the outbreak, FeLV vaccination of all non-viremic captured free-living and captive lynx was implemented [46]. Since regulated necropsies of dead lynx were implemented, other pathogens have been shown to contribute to mortality of this species, including CDV [47], ADV [48,49], *M. bovis* [43], and *Streptococcus canis* [50]. Such findings have spurred many other studies on carnivore diseases in Portugal and southern Spain to provide a more comprehensive characterization of the pathogens circulating in areas of lynx reintroduction [49,51,52].

The Iberian wolf has also been considerably studied over recent years in Portugal and northern Spain. Canine adenovirus-1 (CAdV-1) and CPV-1 are most likely enzootic in Iberian wolf populations [53,54,55]. Our quantitative analysis detected a higher exposure to CPV-1 in the areas where the wolf is more abundant, which may have had influence in shaping this pattern. In contrast, variable rates of exposure have been reported for CDV [53,54,55,56,57,58]. This suggests epizootic behavior dependent on spillovers from dogs [54,58]. Different serological techniques used in these studies may have affected such differences.

Another endangered carnivore that recent evidence suggests is suffering from an infectious disease is the Cantabrian brown bear. CAdV-1 was the proven cause of death in three endangered brown bears [59]. The origin of these cases is so far unknown and might be sympatric wolves or rural dogs. CAdV-1 appears to be enzootic in the sympatric Iberian wolf population [54]. This is another example of the relevance of disease surveillance for the conservation of threatened species and highlights how little we know about the actual impact of diseases in the population dynamics of most species.

Regarding the study of carnivores as reservoirs and/or sentinels of pathogens, many publications in Spain have examined national, regional, or multi-regional scales of infection. Large numbers of carnivores of diverse species were analyzed for microparasites [60,61,62,63,64,65,66,67,68], providing insights into the factors favoring the presence, distribution, and prevalence of pathogens of human, livestock, and domestic pet health (see below). Most if not all these studies were cross-sectional and, regrettably, lacked continuity to ascertain the actual importance of these hosts in the epidemiology of the studied pathogens.

### 4.4. Pathogen-Specific Discussion

#### 4.4.1. Canine Distemper Virus

**Figure 8:**
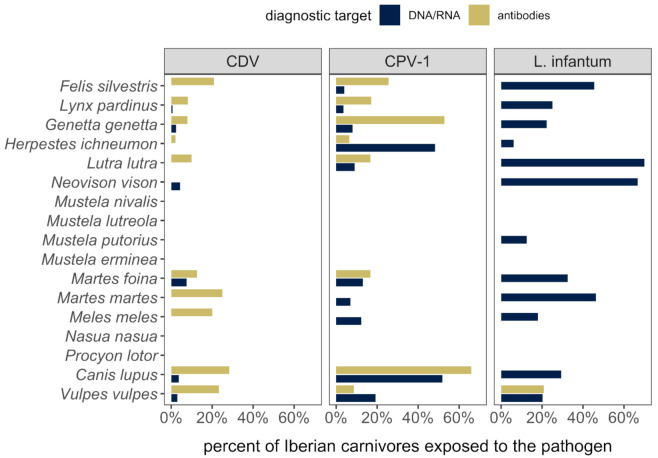
Percent of Iberian carnivores exposed to each of the three most frequently studied pathogens in Iberian carnivores (CDV, CPV-1, Leishmania infantum), stratified by each host species and diagnostic target.

CDV is one of the most frequently investigated pathogens in Iberian carnivores. Exposure and infection is clearly lower than the other equally common CPV-1 (Figure 8), probably owing to CDV having higher pathogenicity and mortality rates and the need for close contact between hosts for transmission. Infection has been demonstrated only in a handful of individuals, but this may be affected by the more rapid degradation of RNA when compared with DNA (for CPV-1), especially from passive surveillance, which is a clear handicap for obtaining PCR products and/or readable sequences. The rarity of molecular characterization of CDV isolates in Iberian carnivores likewise is a huge handicap for understanding the epidemiology of CDV in Iberia. Few molecular characterizations indicated a domestic dog origin [47,69]. Greater CDV exposure in canids identified by our meta-analysis is not surprising given that foxes and wolves are closely related to dogs. In contrast, the greater exposure among mustelids, also pointed out by our quantitative analysis, is somewhat unexpected but may suggest that the broader Caniformia may have some particular susceptibility to CDV [39].

#### 4.4.2. Carnivore Protoparvovirus-1

Sixteen studies have evaluated exposure and/or presence of viral DNA of CPV-1 in over half of all Iberian carnivore species (Figure 8). We found that CPV-1 exposure varied most by geography and was greatest in bioregions 1 and 2, which correspond to the more humid areas of Spain (but might be biased by being the region were wolf studies were performed, as abovementioned). This result likely stems from the ability of CPV-1 to survive for long periods in wet environments [70]. This, combined with the scent-marking behavior of carnivores, facilitates virus transmission.

Of all viruses present in Iberia, the protoparvoviruses have the most data on molecular epidemiology. Combining the different studies from our review, up to 31 PV sequences of the *vp2* gene of variable length from eight carnivore species are available [51,67,71]. In wolves, three canine parvovirus variants have been found, although CPV-2c appears to be the dominant strain currently circulating in this species. In the red fox, the most commonly detected strain was, surprisingly, feline panleukopaenia virus (FPLV). FPLV has been detected not only in other Caniformia, such as the badger and stone marten (although canine strains are more common in both species), but also in Feliformia (genet and mongoose). Conversely, CPV variants such as CPV-2c were confirmed in a wildcat and a genet. Many of these sequences were closely related or equal to sequences from domestic dogs and cats. In addition, CPV-1 exposure in wolves was associated with anthropogenic habitats, which could suggest a role for dog–wolf transmission [55]. These results point out a complex, multi-host pathogen system with frequent cross-species transmission, as observed for this pathogen in other carnivore assemblages worldwide [56].

#### 4.4.3. *Leishmania infantum*

*Leishmania infantum* is probably the most important zoonotic parasite present in the Iberian Peninsula that can be hosted by carnivores. Indeed, the first microparasite to be studied in an Iberian wild carnivore was this protozoan species [34], and it has remained one of the most studied pathogens (Figure 8). Most if not all of the studied carnivore species are susceptible to the parasite. In particular, the red fox is a suitable reservoir due to its ubiquity, abundance, proximity to humans, and the absence of clinical signs associated with this parasite. Nevertheless, experimental evidence regarding enhanced parasite competence is lacking. Although early studies suggested a sylvatic cycle of *L. infantum* in Iberia [61,72], more recent studies indicate that strains circulating in wild carnivores are shared with humans and dogs [73,74]. Leishmaniasis is among the more concerning diseases of dogs in Mediterranean areas. Accordingly, our meta-analysis indicated that sampling effort for *L. infantum* was greatest in canids, but exposure was greater not only in canids but also in mustelids. The latter result is intriguing and clearly calls for more studies about *Leishmania* in mustelids. Although some species such as the stone marten and badger are widespread in Iberia and can be locally abundant [13,14], their role as reservoir hosts for this pathogen is probably negligible or understudied. Nevertheless, mustelids can suffer from clinical leishmaniasis [75].

A remarkable event regarding this parasite is that it was recently demonstrated to be widespread in carnivores in areas that not long ago were considered unsuitable for its sandfly vector, specifically the northwestern part of the Iberian Peninsula [53,73]. This indicates that the expansion of *L. infantum* probably has been driven by vector range shifts affected by global warming and further confirms the usefulness of carnivores as sentinels of pathogen spread.

#### 4.4.4. Piroplasmids

The Iberian Peninsula is the epicenter of two parasitological conundrums regarding two piroplasmids, namely *Babesia vulpes* and *Cytauxzoon* spp. *Babesia vulpes* [76] was first described from a diseased dog that acquired the infection during the Pyrenean region of Spain [77]. This parasite was subsequently confirmed in Spanish dogs [78] and was then found to be prevalent in red foxes from Portugal, Spain [79,80,81], and many other European locations (see revision in [82]). Since this parasite is highly pathogenic for dogs, the high prevalence commonly observed in healthy foxes strongly supports the role of the red fox as the natural host for this parasite.

Until 2004, *Cytauxzoon* was a genus of feline piroplasmdis never reported in Europe until it was found in a Spanish cat [83], a species in which this parasite is very pathogenic. Soon after that, Luaces et al. [84] reported the first case in a European wild feline in an Iberian lynx sampled in 2003. Subsequently, this parasite was found to be prevalent in Iberian lynx, but only in one of its metapopulations, Sierra Morena [84,85,86]. This points to an interesting unknown, which is why the parasite is absent from the other metapopulation (Doñana); the absence of its tick vector, which remains unidentified, may explain this problem. Moreover, *Cytauxzoon* has been found in European wildcats (*Felis silvestris silvestris*) in Sierra Morena [52] and in the northern Basque Country [87]. The identity of the species of *Cytauxzoon* parasitizing lynxes has been under debate. First molecular characterizations of Iberian lynx isolates soon revealed they were closer to a *Cytauxzoon* reported in a Palla’s cat (*Otocolobus manul*) from Mongolia than to *Cytauxzoon felis* and was probably a different species [85]. Recently, and after the finding of *Cytauxzoon* parasites in many other European countries, both in cats, wildcats, and Eurasian lynx (*Lynx lynx*), lead to the establishment of three new *Cytauxzoon* species in Europe [88]. Unfortunately, these characterizations did not include Iberian isolates, and the identity of the *Cytauxzoon* infecting Iberian lynx and wildcats remains to be elucidated.

#### 4.4.5. Sarcocystidae

Carnivores are key hosts in the life-cycle of many parasites of the family Sarcocystidae. For example, *Toxoplasma gondii* is an important zoonotic agent for which all felids can be definitive hosts; conversely, the wolf can be the definitive host for *Neospora caninum*. Nevertheless, other carnivores, even if they cannot shed *T. gondii* oocysts, can be important because they can spread the parasite long distances and because repeated contact with the parasite through food items can lead to atypical strains of the parasite, which can be more pathogenic than classic ones [89]. Serologic surveys carried out in diverse parts of Iberia showed that these parasites are widespread [60,90,91,92,93,94]. The only molecular characterization of an Iberian wild carnivore isolate was carried out by Calero-Bernal et al. [95], who found that Type III was the most frequent variant of *T. gondii* in red foxes. Mixed infections, possibly due to reinfections, were also detected in foxes by Calero-Bernal et al. [95]. No information on any impact of toxoplasmosis in the health of Eurasian otters [96] and other wild carnivores is available in Iberia.

For *Besnoitia besnoiti*, an important cattle pathogen, the definitive host remains unknown, though an undetermined wild carnivore has been proposed to be involved in the parasite life cycle. In the search for this unknown host, Millán et al. [64] analyzed more than 160 serum samples from wild carnivores from 14 species with negative results. González-Barrio et al. [97] analyzed fecal samples from 12 carnivores, obtaining sequences closely related to *B. besnoiti* in red foxes, resulting in the first detection in a fecal sample of a wild carnivore worldwide. Nevertheless, the lack of complementary techniques to confirm this finding and the fact that genomic material found in the feces of predators can be part of their diet limits the interpretation of these results.

#### 4.4.6. Selected Bacteria

*Mycobacterium bovis* is enzootic in wild ungulates in Iberian Mediterranean ecosystems [98]. This exposes carnivores to this bacterium during predation or scavenging. Positive cultures not associated with macroscopic lesions are usually found in carnivores [99], but lesions and even disseminated disease were observed in lynx [43,100] and red foxes [41,101,102]. Spoligotyping performed in Doñana National Park revealed that the same strains were shared by lynx, red foxes, and wild ungulates [103]. In Northern Spain, the badger has attracted attention of researchers because this species is the main reservoir of *M. bovis* in the British Islands. Nevertheless, the prevalence in Northern Spain was found to be lower than that in Britain [104], probably because badgers from Northern Spain do not form large social groups as their British counterparts do. Iberian carnivores can be also frequently exposed to another mycobacterium, *M. avium paratuberculosis* [105,106]. Positive cultures were obtained from 10% of foxes, 6% of mongooses, and other carnivores without observing macroscopic lesions, suggesting these species act as subclinical carriers. Carnivores have also been identified as asymptomatic carriers of *Salmonella* sp. [107] and are exposed to various pathogenic *Leptospira*, including serovars typical from rodents, cattle, and dogs [42,94,108,109].

## 5. Pitfalls and Perspectives

Iberian carnivores have been investigated for a large number of relevant pathogens, but most of these were studied in one or two publications. Furthermore, the majority of studies identified in our systematic review were cross-sectional, with a clear lack of longitudinal studies of infectious disease in Iberian carnivores. Such studies are necessary to identify temporal infection dynamics, their drivers (e.g., climate, reproductive seasonality, resource scarcity, etc), and, when linked to population data, the true impact of microparasites on population dynamics [110]. We also identified the following pitfalls and concerns in the study of infectious diseases of Iberian carnivores, which should be addressed by researchers in future work:

- Transnational collaboration is necessary for endangered carnivore species that share territories across Spain and Portugal, such as the Iberian lynx and wolf. There are no major physical barriers between these two countries to limit the exchange of carnivore species and the pathogens they share.
- Systematic necropsies and histopathological studies of dead or sick carnivores are essential to understand whether pathogens induce disease in individuals e.g., [111], having the potential for impact at the population level. Collaboration among wildlife recovery centers and researchers and pathologists are highly recommended in this regard.
- Researchers should consider approaching their studies in a coordinated way, sharing samples, and applying the same diagnostic techniques. Diagnostic techniques, especially serological ones, should be validated for each individual species. Otherwise, results must be interpreted carefully.
- It is crucial to determine the species (one of more) of *Cytauxzoon* circulating in domestic cats, wildcats, and Iberian lynx to determine whether these species are shared among them.
- Molecular characterization of important apicomplexans such as *T. gondii* and *N. caninum* are crucial to uncover the strains circulating in Iberian wild carnivores and whether or not these serve as reservoirs of new variants and/or of variants implicated in disease in humans and livestock.
- Identifying the vectors for *B. vulpes* and *Cytauxzoon* sp. is basic epidemiological and preventive knowledge, especially considering that these parasites are highly pathogenic for domestic dogs and cats, respectively.
- It is important to increase our knowledge about the drivers of cross-species transmission, with emphasis on those more virulent pathogens, such as CDV, for which little molecular information is available. This prevents understanding the epidemiology and population impacts of canine distemper in Iberian wildlife.
- More studies of urban carnivores are required. Urban and periurban environments have higher possibilities of wild carnivore contact with humans and their dogs and cats and in turn higher chances of pathogen spillover e.g., [112]. Although some approaches have been done in this regard in Iberia [81], there is ample remaining work to do on the urban ecology of microparasites in Iberian carnivores.
- We lack information about whether protozoans such as *B. vulpes*, *Cytauxzoon* sp., *H. canis* and especially *L. infantum* cause clinical manifestations in the parasitized carnivores [75,113] or have any relevance at the population level. Although lack of pathogenicity is commonly assumed by researchers due to the absence of apparent lesions during captures or necropsies, more detailed studies are necessary because alterations may not be recognized at the macroscopic level.
- Although we have identified many pathogens to be assessed in Iberian carnivores, there is no information about some other pathogens that may be causing unrecorded disease, such as *Hepatozoon* sp., *Brucella canis*, *Francisella tularensis*, or the recently described feline morbilliviruses, which are prevalent in wild felines in other regions of the world [114].
- Fungal pathogens have largely been neglected to date. Although *E. bieneusi* was detected in red fox fecal samples [32], and an unpublished document reported infection with *Coccidiodes immitis* in a badger in Northern Spain [115]. This and other fungal pathogens such as *Histoplasma* spp. [116] have not been investigated in detail.
- Our meta-analysis confirmed that the Arctoidea are underrepresented in Iberian carnivore research. The infectious diseases of the brown bear have been clearly understudied, apart from the above-mentioned known mortalities due to canine infectious hepatitis and a case of clostridiosis caused by *Clostridium sordellii* [117]. No information is available from the Pyrenean sub-population in particular. Very little is known for another threatened carnivore, the European mink [118]. The threatened status of these species necessitates more comprehensive information about their infectious diseases and their population impacts.
- Other members of the Arctoidea, i.e., the small-sized mustelids such as the stoat, the weasel, and the polecat, have also received little attention. Presumably, this sampling bias is driven by their small size, difficulty to detect or capture, or some lack of interest from researchers. More studies of these species are necessary to provide a more comprehensive picture of pathogen diversity in Iberia.

In conclusion, our understanding of infectious diseases in Iberian wild carnivores has significantly advanced in the last twenty years, and this increased information has prompted new research questions and exciting future directions. We encourage more coordinated work among different Iberian research groups and for open data sharing that will allow future integrative and meta-analytic insights into the drivers of pathogen exposure, population impact, and zoonotic risk in wild Iberian carnivores.

## Figures and Tables

**Table 1:** Iberian carnivores. Distribution refers to the bioregions displayed in Figure 1.

Family/Species	Origin	Bioregions	Observations
**Suborder Caniformia**			
**Family Canidae**			
Wolf (*Canis lupus*)	Endemic	All	About 2000 individuals. Not evenly distributed (mostly in 1, 2, 6)
Fox (*Vulpes vulpes*)	Endemic	All	Legally hunted in Spain and Portugal
**Family Mustelidae**			
Eurasian badger (*Meles meles*)	Endemic	All	
Pine marten (*Martes martes*)	Endemic	1, 2, 5, 6	Present in some of the Balearic Islands
Stone marten (*Martes foina*)	Endemic	All	Present in some of the Balearic Islands
Stoat (*Mustela erminea*)	Endemic	1, 2, 5	Only in the northernmost part of Iberia
European mink (*Mustela lutreola*)	Endemic	1, 2	Less than 500 indivuals
Western polecat (*Mustela putorius*)	Endemic	All	
Least weasel (*Mustela nivalis*)	Endemic	All	Present in some of the Balearic Islands
American mink (*Neovison vison*)	Introduced (recent)	1, 2, 4, 5	
Eurasian otter (*Lutra lutra*)	Endemic	All	
**Family Ursidae**			
Brown bear (*Ursus arctos*)	Endemic	1, 2	About 250 individuals in (1) and 50 in (2)
**Family Procyonidae**			
Raccoon (*Procyon lotor*)	Introduced (recent)	4	Occasional detections all around the peninsula and Mallorca Island.
South American coati (*Nasua nasua*)	Introduced (recent)	6	Recently introduced in Mallorca Island
**Suborder Feliformia**			
**Family Viverridae**			
Common genet (*Genetta genetta*)	Introduced (historical)	All	Present in some of the Balearic Islands
**Family Herpestidae**			
Egyptian mongoose (*Herpestes ichneumon*)	Introduced (historical)	3, 5, 6	Legally hunted in Portugal
**Family Felidae**			
European wildcat (*Felis silvestris silvestris*)	Endemic	All	
Iberian lynx (*Lynx pardinus*)	Endemic	3, 5, 6	About 1000 individuals, 60% in (3).

## Data Availability

Data is available at https://data.mendeley.com/drafts/5y5d8b4hzc.

## Supplementary Materials

The following are available online at https://www.mdpi.com/article/10.3390/ani11092708/s1, Figure S1. Phylogenetic correlation matrix of the 18 Iberian carnivore species included in our data. Figure S2. Predicted infection prevalence from pathogen taxon-specific meta-analysis models. Figure S3. Predicted seroprevalence from pathogen taxon-specific meta-analysis models. Figure S4. Predicted exposure positivity from key pathogen-specific meta-analysis models. Figure S5. Taxonomic patterns in pathogen-specific prevalence across Iberian carnivores. Figure S6. Modeled relationship between carnivore phylogenetic distance and pathogen sharing. Table S1. Post-hoc comparisons from the Poisson GLM of sampling effort for carnivore family. Table S2. Post-hoc comparisons from the Poisson GLM of sampling effort for bioregion. Table S3. Post-hoc comparisons from the negative binomial GLM of pathogen richness for carnivore family. Table S4. Post-hoc comparisons from the negative binomial GLM of pathogen richness for bioregion. Table S5. Predicted infection prevalence from our meta-analysis model. Table S6. Predicted seroprevalence from our meta-analysis model. Table S7. Results of phylogenetic factorization applied to each primary response variable. Table S8. Test statistics from phylogenetic meta-analysis models applied to infection prevalence and seroprevalence data. Table S9. Predicted infection prevalence from our meta-analysis models for each broad pathogen taxon. Table S10. Predicted seroprevalence from our meta-analysis models for each broad pathogen taxon. Table S11. Test statistics from phylogenetic meta-analysis models applied to exposure for the three focal pathogens. Table S12. Results of phylogenetic factorization applied to prevalence or seroprevalence for each main pathogen taxon. Table S13. Results of phylogenetic factorization applied to prevalence or seroprevalence for the three most common pathogens in Iberian carnivores. Table S14. Results from the negative binomial GAM predicting pathogen sharing. Table S15. Post-hoc comparisons from the negative binomial GAMM of pathogen sharing.
