# Supplementary figures and images for: Patterns of Exposure and Infection with Microparasites in Iberian Wild Carnivores: A Review and Meta-Analysis
Source: Animals (Basel). 2021 Sep 16;11(9):2708. doi: 10.3390/ani11092708 (PMC8469010; doi:10.3390/ani11092708)

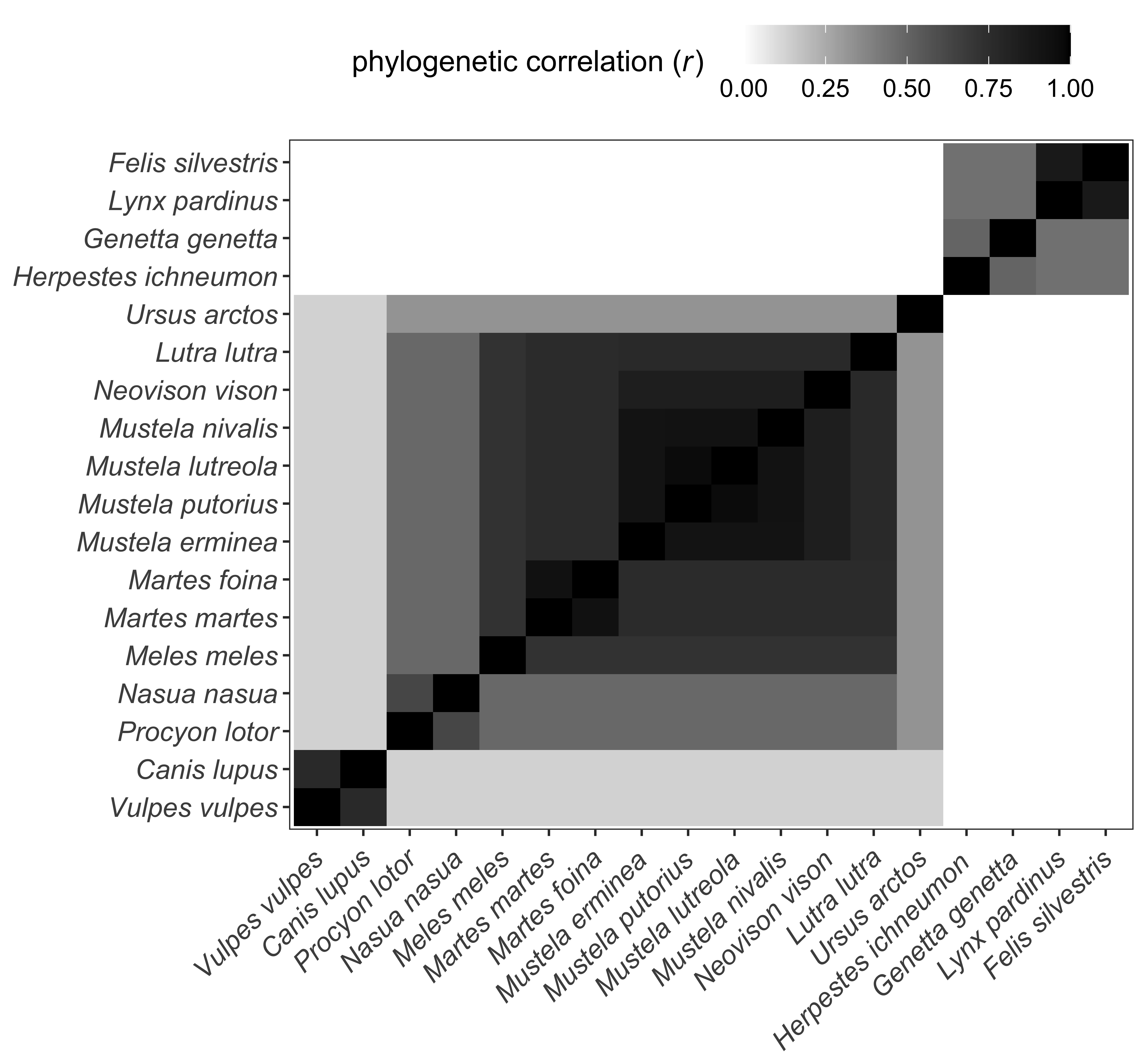

Supplement: Supplementary file 1 [file animals-11-02708-s001.zip › Figure S1.png]

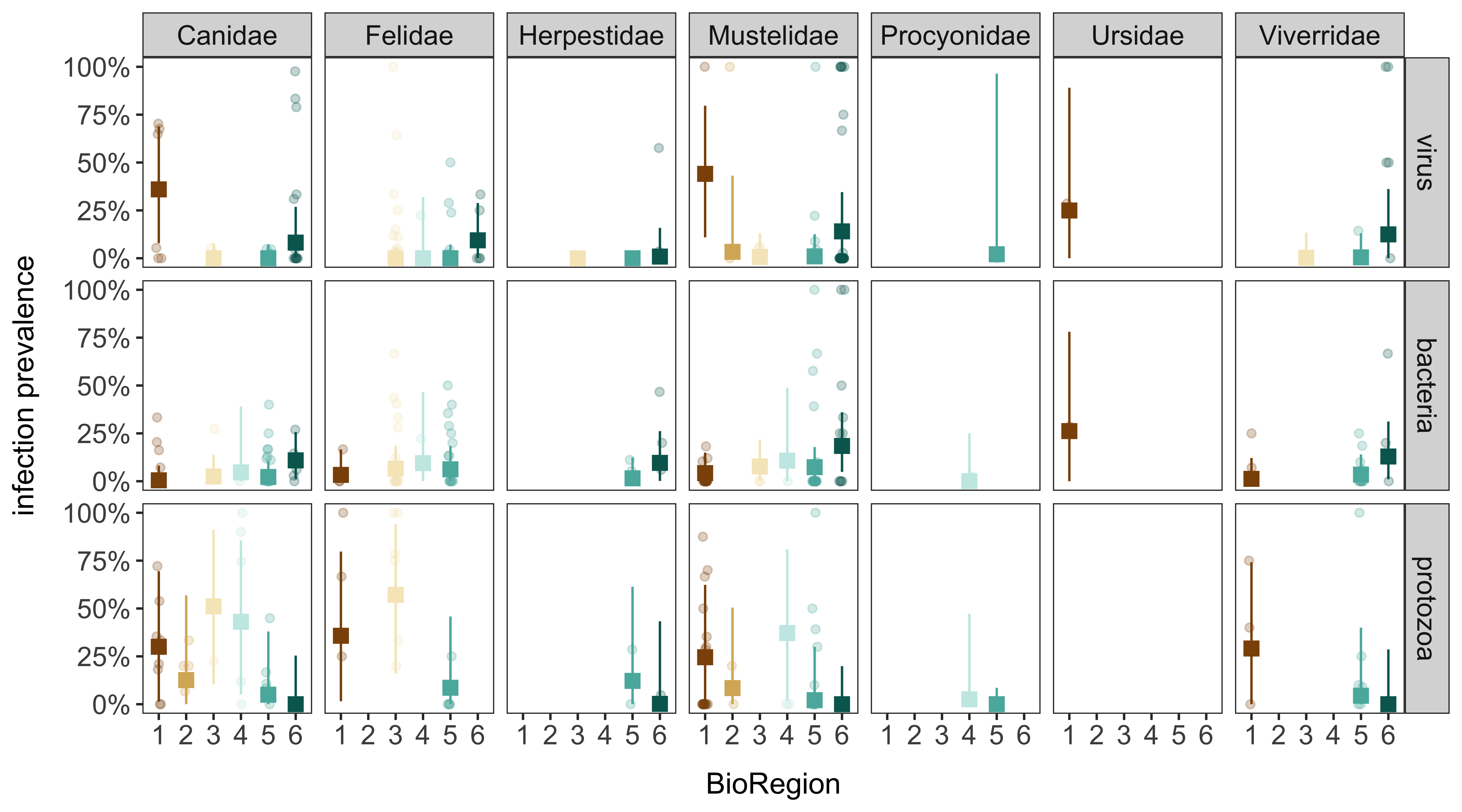

Supplement: Supplementary file 1 [file animals-11-02708-s001.zip › Figure S2.png]

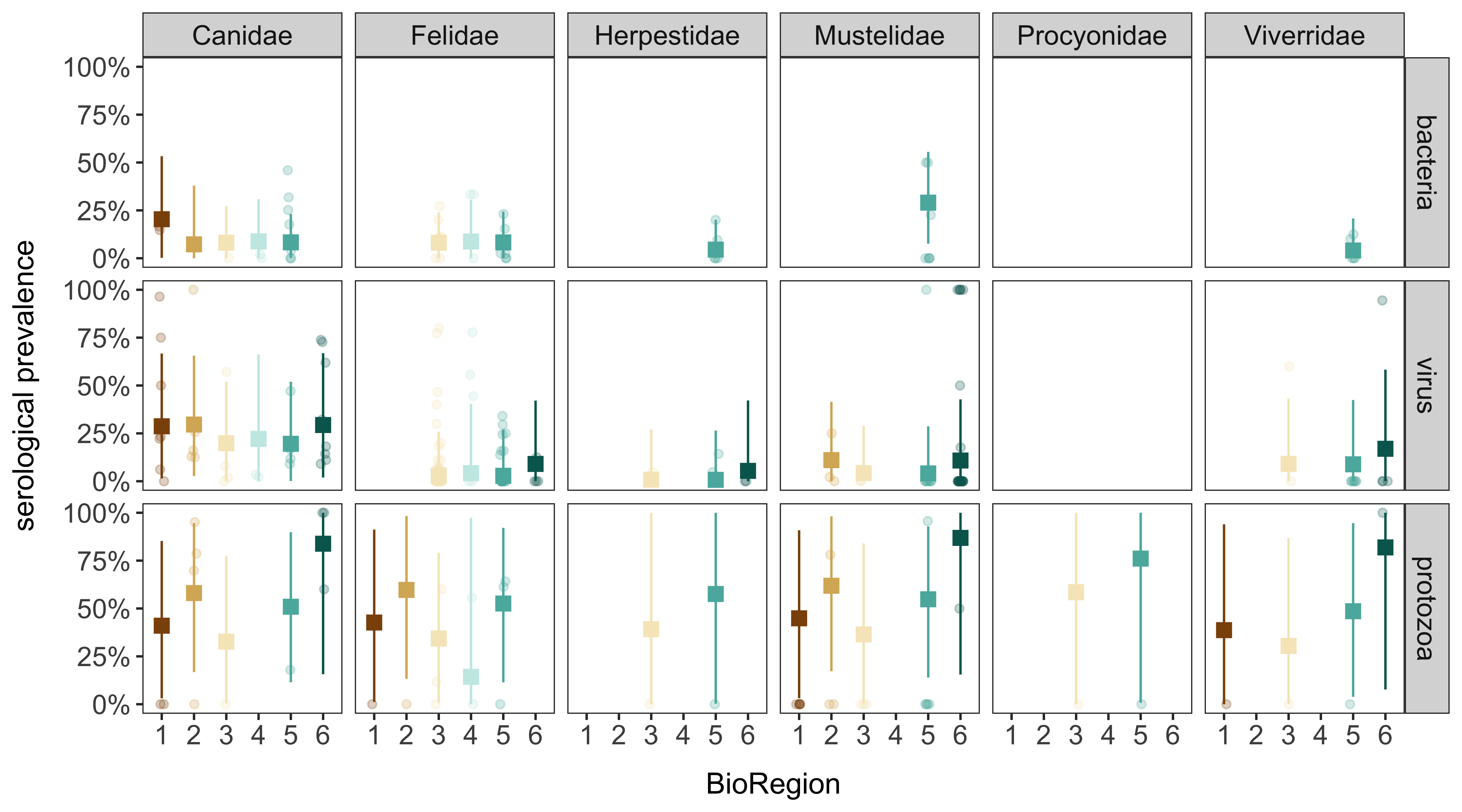

Supplement: Supplementary file 1 [file animals-11-02708-s001.zip › Figure S3.png]

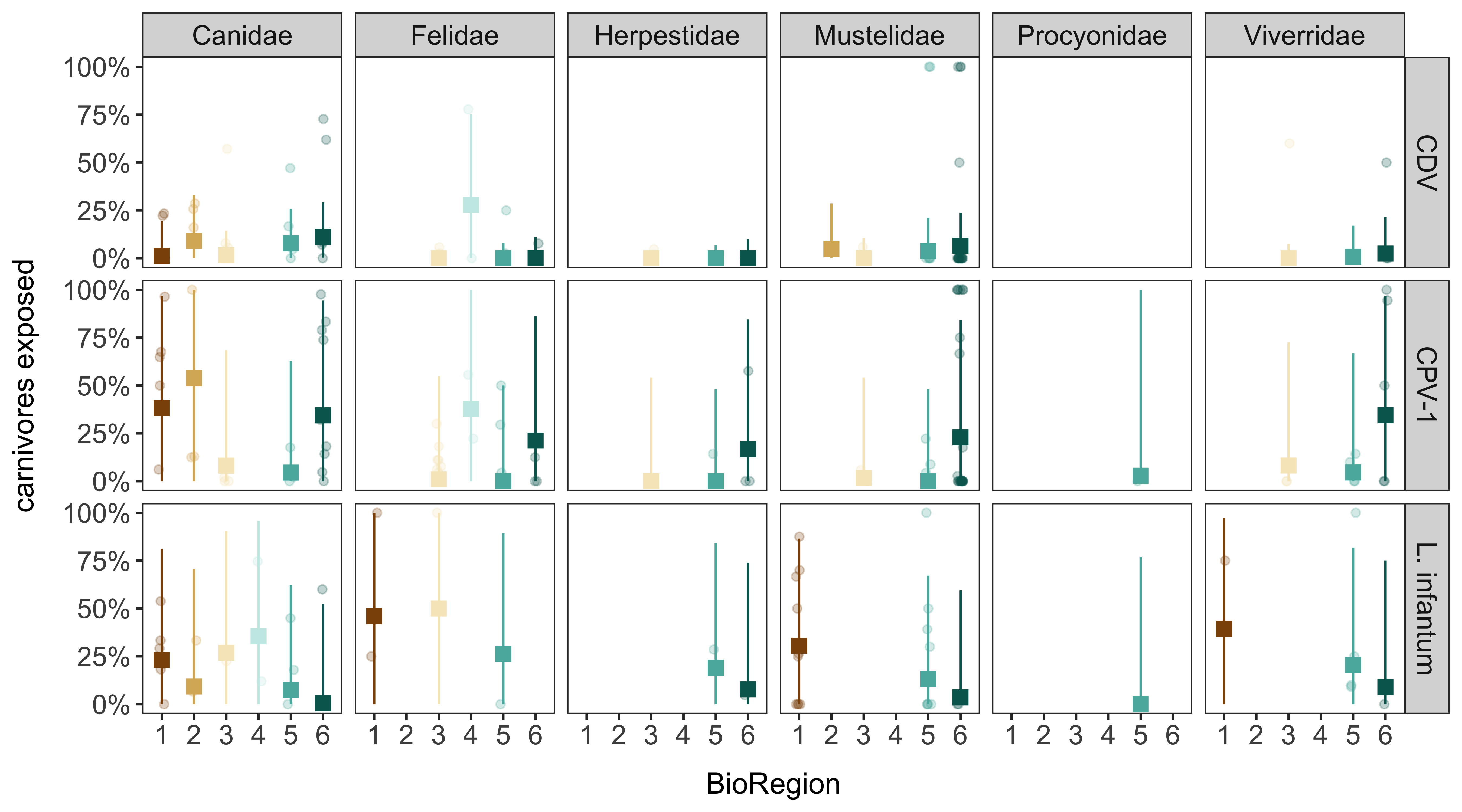

Supplement: Supplementary file 1 [file animals-11-02708-s001.zip › Figure S4.png]

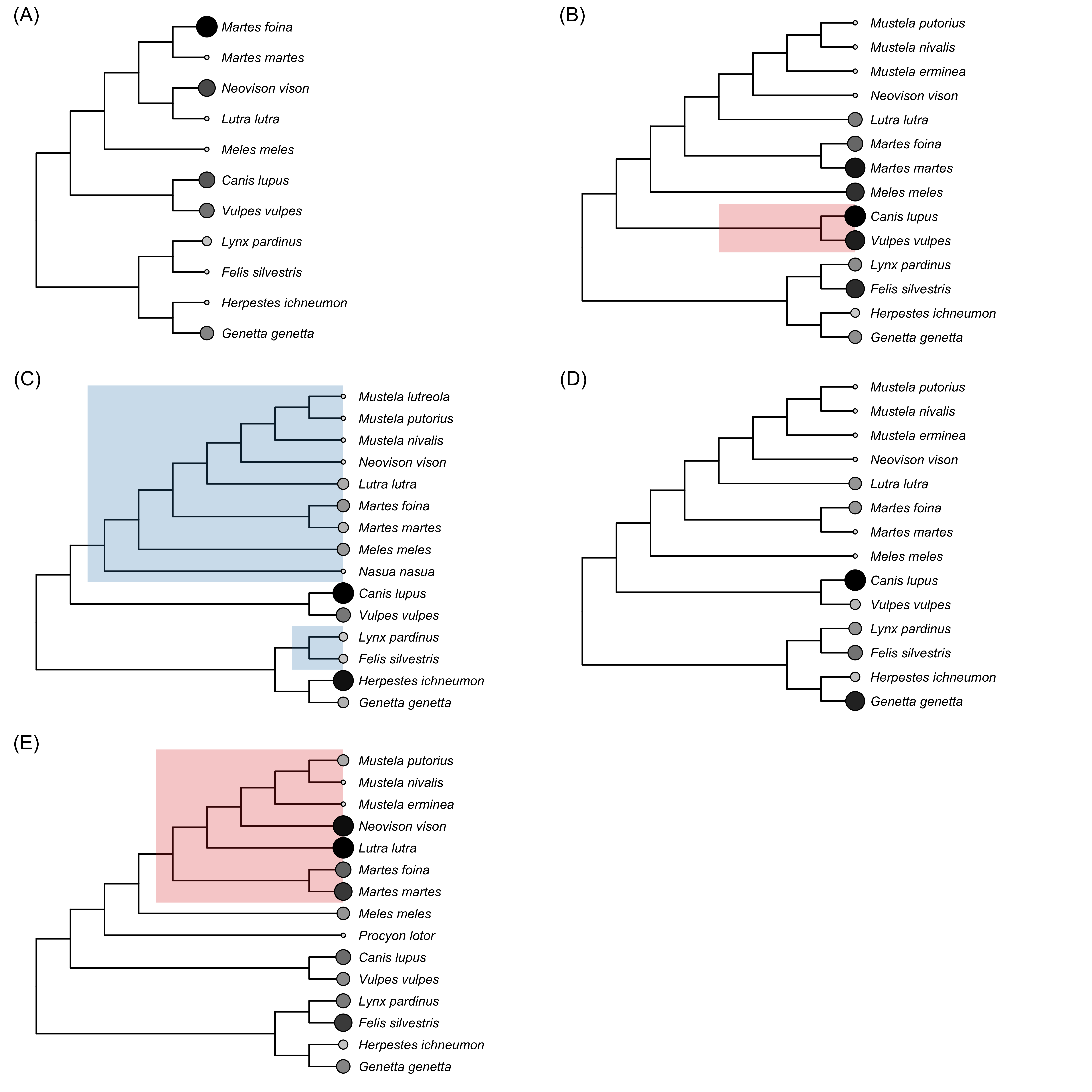

Supplement: Supplementary file 1 [file animals-11-02708-s001.zip › Figure S5.png]

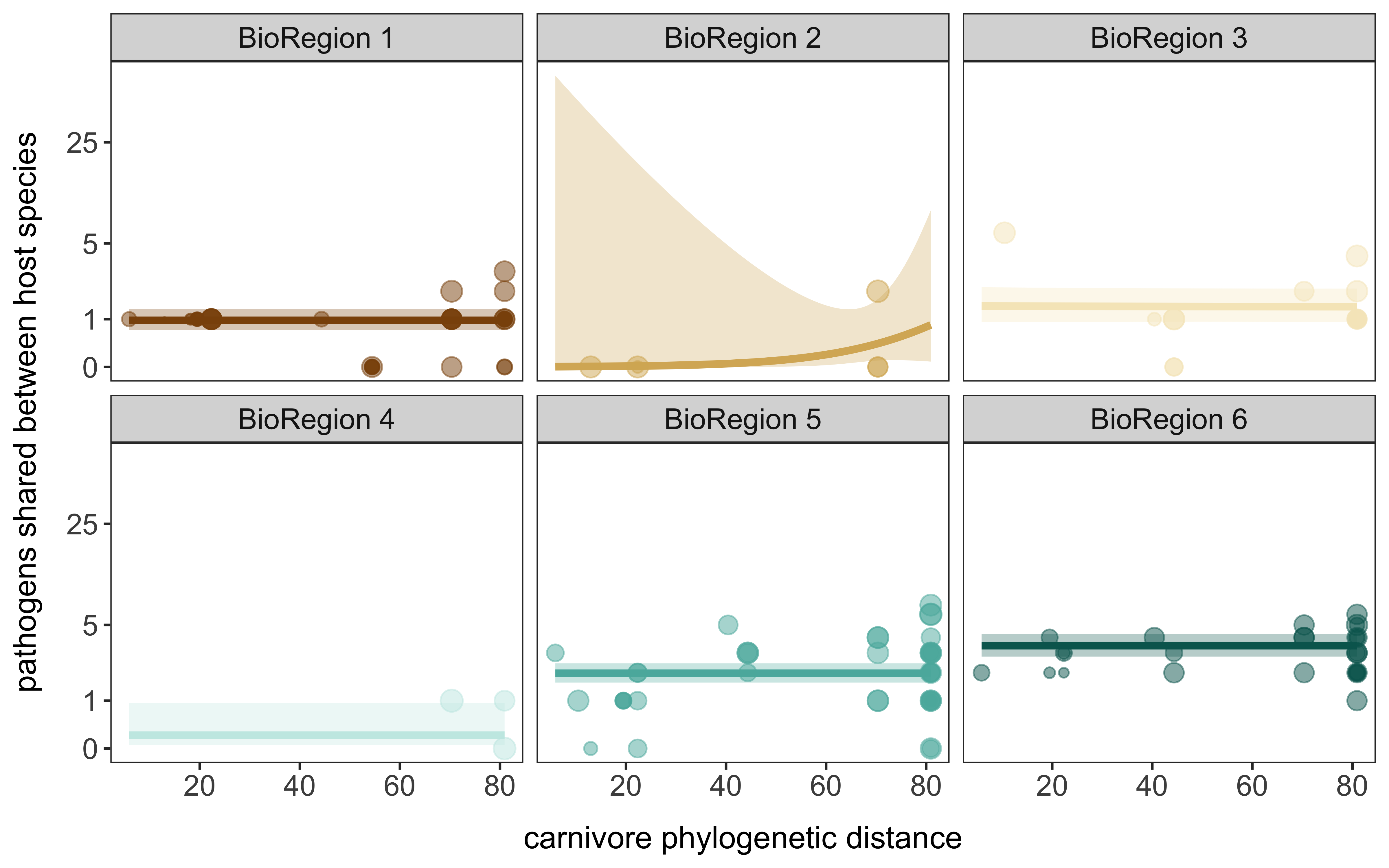

Supplement: Supplementary file 1 [file animals-11-02708-s001.zip › Figure S6.png]
